# Phone-based Intervention under Nurse Guidance after Stroke (PINGS): study protocol for a randomized controlled trial

**DOI:** 10.1186/s13063-016-1557-0

**Published:** 2016-09-05

**Authors:** Fred Stephen Sarfo, Frank Treiber, Carolyn Jenkins, Sachin Patel, Mulugeta Gebregziabher, Arti Singh, Osei Sarfo-Kantanka, Raelle Saulson, Lambert Appiah, Eunice Oparebea, Bruce Ovbiagele

**Affiliations:** 1 Division of Neurology, Department of Medicine, Kwame Nkrumah University of Science and Technology, P.M.B., Kumasi, Ghana; 2 Komfo Anokye Teaching Hospital, Kumasi, Ghana; 3 Technology Applications Center for Healthful Lifestyles, Medical University of South Carolina, South Carolina, Ghana; 4 Department of Nursing, Medical University of South Carolina, South Carolina, Ghana; 5 Department of Psychiatry, Medical University of South Carolina, South Carolina, Ghana; 6 Department of Public Health Sciences, Medical University of South Carolina, South Carolina, Ghana; 7 Department of Neurology, Medical University of South Carolina, South Carolina, Ghana

## Abstract

**Background:**

Hypertension is the premier modifiable risk factor for recurrent stroke. In sub-Saharan Africa (SSA) where the stroke burden is escalating, little is known about the role of behavioral interventions in enhancing blood pressure (BP) control after stroke.

Our objective is to test whether an m-Health technology-enabled, nurse-led, multilevel integrated approach is effective in improving BP control among Ghanaian stroke patients within 1 month of symptom onset compared with standard of care.

**Methods:**

This two-arm cluster randomized controlled feasibility pilot trial will involve 60 recent-stroke survivors. Using a computer-generated sequence, patients will be randomly allocated into four clusters of 15 patients each per physician: two clusters in the intervention arm and two in the control arm. Patients in the intervention arm will receive a simple pillbox, a Blue-toothed UA-767Plus BT BP device and smartphone for monitoring and reporting BP measurements and medication intake under nurse guidance for 3 months. Tailored motivational text messages will be delivered based upon levels of adherence to the medication intake. Both groups will be followed up for 6 months to compare BP control at months 3, 6 and 9 as primary outcome measure. Physicians assessing BP control will be blinded to arms into which patients are allocated. Secondary outcome measures will include medication adherence scores and Competence and Autonomous Self-regulation Scale scores. A qualitative study is planned after follow-up to explore the lived experiences of participants in the intervention arm.

**Discussion:**

A feasible and preliminarily effective intervention would lead to a larger more definitive efficacy/effectiveness randomized controlled trial powered to look at clinical events, with the potential to reduce stroke-related morbidity and mortality in a low- to middle-income country.

**Trial registration:**

ClinicalTrials.gov Identifier: NCT02568137, registered on 13 July 2015.

**Electronic supplementary material:**

The online version of this article (doi:10.1186/s13063-016-1557-0) contains supplementary material, which is available to authorized users.

## Background

Hypertension (HTN) is the premier modifiable risk factor for recurrent stroke [1]. Fortunately, with control of HTN [2], recurrence of and mortality from stroke can be greatly reduced [3–5] as has been seen in several high-income countries (HICs) [6–9]. However, for low- and middle- income countries (LMICs) that disproportionately bear the global burden of stroke, these gains have not materialized [10]. Data from the Global Burden of Disease 2013 study suggest that deaths and disability-adjusted life years from stroke in LMICs account for 75.2 % and 81.0 % of global estimates, respectively [11].

Achieving and sustaining blood pressure (BP) control is a global challenge [12] particularly in LMICs including sub-Saharan Africa (SSA) [12–14]. A multinational survey has identified that 46.5 % of participants with HTN were aware of the diagnosis and 32.5 % of hypertensive participants had their BP controlled, with rates of awareness and control of BP significantly higher in HICs compared with LMICs [12]. Key factors responsible for uncontrolled HTN are medication non-adherence and failure to intensify therapy in a timely manner (i.e., therapeutic inertia) [15, 16]. Furthermore, the lack of availability, poor affordability and low rates of utilization of secondary prevention cardiovascular medicines in LMICs [17, 18] combined with low literacy levels, traditional beliefs and misconceptions about HTN may all contribute to the low prevalence of HTN awareness, treatment and control which poses a serious threat to stroke prevention.

Systematic reviews of randomized controlled trials (RCTs) involving uncontrolled hypertensive patients have indicated that BP self-monitoring, medication-reminding tactics and the use of case managers each improve adherence, therapeutic inertia and BP levels [19–22]. No RCTs looking at medication adherence or BP self-monitoring have been designed specifically for people in SSA, especially for those at high risk for future stroke. However, mobile health (m-Health) technology offers a promising approach to address this need [23–30]. Most adults in SSA own a cell phone (approximately 73 %) [31, 32], smartphone ownership is burgeoning (approximately 35 %) [33], and m-Health has produced promising results in chronic disease management (e.g., HIV) in SSA [34–36].

An iterative, theory-guided, design process was used by our group to develop an m-Health BP self-management control program for poor, rurally located Hispanic and African American hypertensive patients called the Smartphone Med Adherence Stops Hypertension: SMASH) [37, 38]. SMASH included multilevel components:

- Patient-level interventions included: (1) sequential automated reminder signals, (2) tailored Short Message Service (SMS)/voice mail motivational and reinforcement messages based upon adherence to daily medication adherence and BP monitoring (every 3 days); and provider-level interventions entailing: (a) emailed summary reports with stepped care guidelines attached every 2 weeks, (b) phone alerts to clinic nurse navigator when verified out-of-range BP measurements occur. Several 3- and 6-month pilot RCTs were conducted with Hispanics and African Americans with uncontrolled HTN. In each RCT, we observed high acceptability, self-efficacy for following a medical regimen, 95–100 % medication adherence, a mean of 95 % achieving guideline-designated BP control compared to an average of 18 % in standard care (SC) patients and large systolic blood pressure (SBP) reductions (mean: -32 mmHg) [39, 40]. SMASH is capable of adaptation to other poor, undereducated patients with HTN using an iterative patient/provider-centered design approach.

The Phone-based Intervention under Nurse Guidance after Stroke (PINGS) study is fashioned after the SMASH program. The objective of the PINGS study is to test whether a theoretical-model-based, m-Health technology-enabled, multilevel integrated approach is feasible in improving sustained BP control among 60 recent Ghanaian stroke patients within 1 month of their stroke, while building capacity in Ghana for longer-term testing of m-Health technology for chronic disease management in this resource-limited setting. A nurse navigator will be employed in the study design as a task-shifting strategy in the care of chronic diseases in SSA where there is a perennial paucity of neurologists and physicians [41].

## Methods

### Trial design

**Fig. 1:**
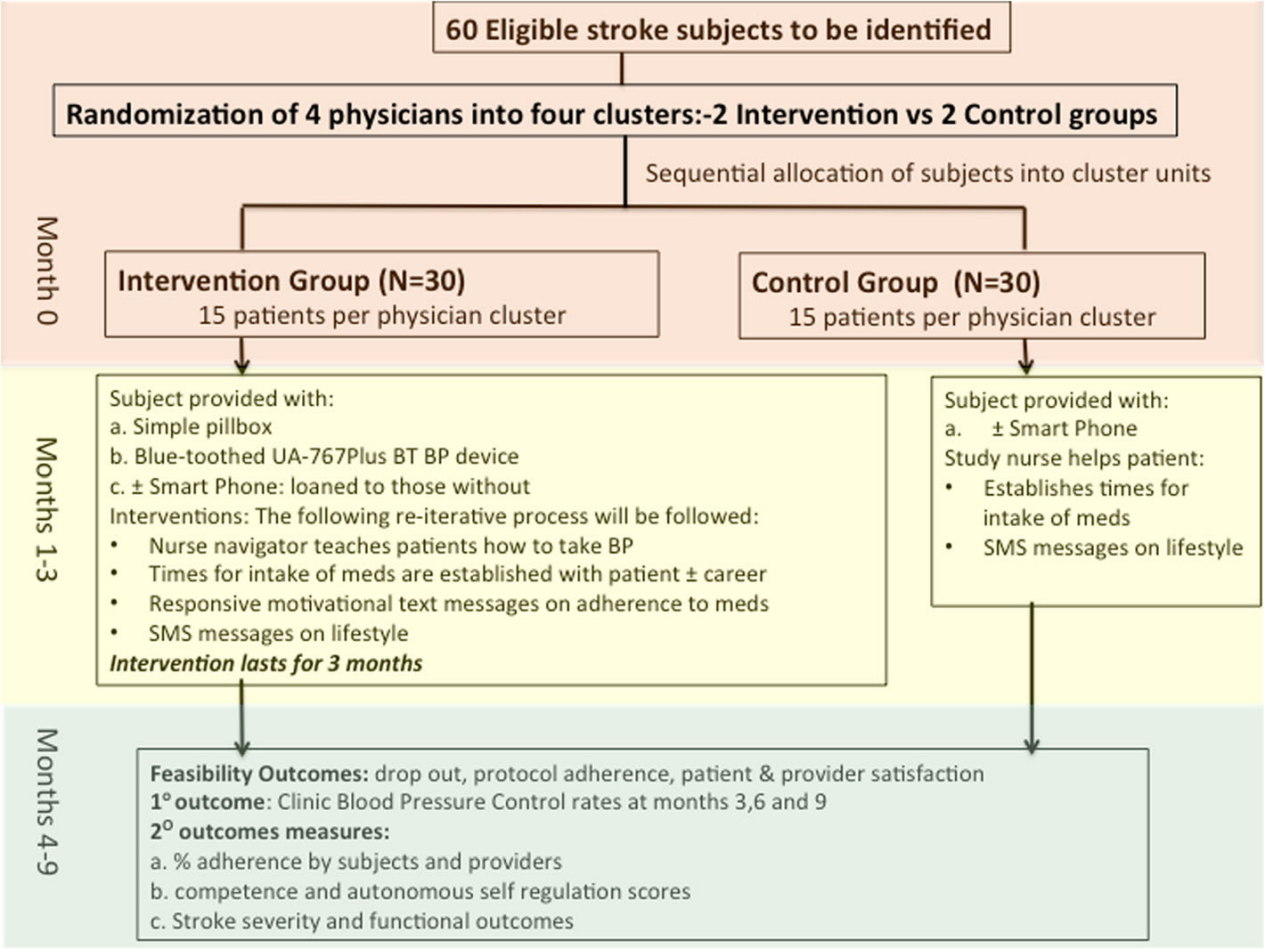
Phone-based Intervention under Nurse Guidance after Stroke (PINGS) study algorithm

This is a two-arm cluster randomized controlled trial involving 60 stroke survivors with the physician as unit of randomization and patients as unit of analysis. Each of the four physicians will be recruited and randomly assigned to the intervention or control group by the statistician (who is not involved in provider or patient recruitment nor data collection) using a computer-generated random sequence. Four groups/clusters of 15 eligible patients will be assigned to either control or intervention arms by the study coordinator after having met study criteria for enrollment (Fig. 1). A cluster randomized trial has been chosen to avoid potential treatment contamination in which physicians might unintentionally provide patients with different degrees of attention or blending of treatment protocols. The checklist for the study protocol is provided as Additional file 1.

### Study setting

This study will be conducted at the Neurology Clinic in Komfo Anokye Teaching Hospital, a tertiary referral center in Kumasi, Ghana [42].

### Study participants

The participants will be 60 adult Ghanaian recent-stroke patients (within 1 month of stroke onset) with uncontrolled HTN who meet the inclusion/exclusion criteria. SBP ≥140 mmHg is used as the selection variable since most patients with HTN who are aged below 65 years have systolic or combination systolic/diastolic hypertension and, for the majority, controlling SBP results in diastolic blood pressure (DBP) also being controlled [43, 44].

### Inclusion criteria

1. Above the age of 18 years; male or female
2. A stroke diagnosis no longer than 1 month before enrollment
3. Have uncontrolled HTN (SBP ≥140 mmHg) based upon last pre-stroke inpatient or outpatient encounter clinic within the previous 12 months, as well as post-stroke encounter and at the screening/recruitment visit
4. Must be legally competent
5. Must own or have a close home-sharing family member with a cell phone

### Exclusion criteria

1. Failure to meet any of the inclusion criteria
2. Severe cognitive impairment/dementia (Modified Mini-Mental State Examination (MMSE) score ≤24)*
3. Severe global disability (modified Rankin Scale (mRS) score ≥3)*
4. Renal dialysis; awaiting renal transplant or transplant recipient
5. Cancer diagnosis or treatment in the past 2 years
6. Planned pregnancy
7. Vulnerable populations, such as pregnant or nursing women, prisoners and institutionalized individuals
  *MMSE score ≤24 and global disability (mRS) score ≥3 excludes patients who have severe cognitive impairments and medical limitations that would interfere with adequate participation in the PINGS project.

### The intervention

#### Patient level

Patients in the intervention group will be given an inexpensive simple pillbox and loaned a Blue-toothed UA-767Plus BT BP device along with a medical regimen assistance application for their smartphone for automatic relay of BP data to a central server at the Medical University of South Carolina. Patients needing a loaner 2 or 3G phone will receive one. The PINGS nurse navigator and patient will establish times that antihypertensive medications and other medications such as antiplatelet agents, statins and oral hypoglycemic agents will be taken daily for entry into the application. The patient (and caregiver) will show that they can properly load the pill tray, view the BP protocol demonstration video and successfully take three consecutive BP measurements using the app’s protocol, and view the feedback chart and/or see their immediate BP data from that session on their phone.

They will receive written and oral information on adherence criteria:

1. To take their medications within 2 hours of designated times,
2. To take their BP every 3 days in the morning and evening

A list of motivational and reinforcement messages will be developed by adapting ones used in earlier m-Health BP control studies [38–40] and enhancing them guided by self-determination theory constructs of competence and autonomous self-regulation [45, 46]. These tailored brief SMS messages will be based on the previous day’s reported medication adherence levels. After two consecutive weeks of 100 % adherence (i.e., medication intake reports using established algorithms based upon proximity to preestablished times for pill intake, using time stamps of when doses were taken, sent via phone) and verification with Medication Possession Ratios (MPRs), the schedule of SMS message delivery will switch to a variable interval schedule [38, 47]. Messages will be tapered to several times per week on a 3-day average variable interval schedule unless adherence drops to below 90 %.

Patients will receive a SMS reminder message on the morning that they are to take their BP measurements. They will receive a tailored SMS message reinforcing their having taken their BP measurements the day after doing so. After each BP session, patients will receive visual BP feedback on their phone, and can select charts showing cumulative averages across weeks/months compared to BP control threshold lines. Patients will also receive lifestyle tips via SMS.

After completion of the 3-month trial, patients will return the BP monitors (and smartphones). Those who experience delayed monthly refill (more than 3 days from the designated time) or uncontrolled BP or a missed appointment on two occasions at the standard 3-month clinic visit will be called by the nurse navigator. The nurse navigator will be trained to conduct motivational interviewing to help these patients identify barriers/issues and develop an action plan. This may include a patient restarting some PINGS’ components (e.g., medication intake reminder alert via SMS delivery system reactivation, motivational messages), being referred for additional help (e.g., mental health), and so forth.

#### Provider level

Physicians in the intervention arm will receive summary reports of their patients’ adherence to their medications and BP data tailored by them (e.g., average and range of SBP/DBP readings, percent of SBP/DBP controlled, and so forth) every 2 weeks using only patient ID numbers. They will also receive summary charts of expert consensus BP management guidelines used in other studies [47]. If a patient’s mean BP exceeds the thresholds, the PINGS nurse navigator will call the patient and conduct the BP protocol again, and initiate a follow-up action as needed. The nurse navigator will also contact those patients who show medication adherence below .80 and/or whose monthly MPRs do not reflect the information in the self-reported medication intake summary reports. They will use motivational interviewing to help the patient to eliminate the barriers related to poor medical regimen adherence.

### Control arm/standard of care

Inclusion criteria include owning a cell phone with at least SMS and voice mail. To control for attention exposure, they will receive SMS messages dealing with healthy lifestyle behaviors (smoking, diet, physical activity) but not with medication adherence, hypertension- or stroke-related issues. Messages will be of the same frequency and size as those in the intervention arm. Every 3 days (comparable to those in the intervention arm) they will receive lifestyle messages and if they have a smartphone, the SMS messages will have either PDFs attached or weblinks to a brief video clip or series of charts, information, and so forth. This information will be sent in two messages and requires 3–5 min per message to view or listen to (similar to the BP protocol duration). All four physicians will receive the expert consensus guideline charts. Intervention tactics will cease for both groups after the 3-month trial.

### Follow-up phase

Subjects in both arms of the study will be followed up for 6 months after the initial 3-month trial. During the follow-up period, subjects will attend clinics monthly where their physicians will assess them clinically. At months 3, 6 and 9 post enrollment patients and providers will undergo assessments on methodological parameters and consumer responses, clinic BP measurements, medication adherence, health literacy on stroke and HTN as well as self-determination theory constructs such as Autonomous Self-regulation and Competence Scales. Physicians in both groups will also undergo assessment on adherence to BP management protocols (Fig. 1 and Table 1).

**Table 1:** Study outcome measures

Outcomes	Psychometrics (internal consistency [α], test-retest reliability [r]) NOTE A^a^	Time points
Primary outcomes		
Feasibility: methodological parameters and consumer responses	Recruitment and retention rates; Self-report: Patient/Provider Satisfaction Scale (.82–.96, test-retest (1 week) .98) [74, 75], fidelity checklists: patient level (e.g., connection; BP uploads via phone; opening of messages/educational information and provider level (e.g., delivery of provider summaries; phone alerts)	Baseline, 3, 6, 9 months
Clinic: BP	Clinic-based BP	
Feedback and refinement	Focus groups	Providers and IG: 9 months
Secondary outcomes		
Medication adherence Physician adherence	Medication Possession Ratio (MPR); Morisky Medication Adherence Scale (α = .76–.83, r = .64) [76, 77], provider guideline adherence: timely med changes (date of script changes following BP feedback (bi-monthly PINGS reports/clinic BPs)	Baseline, 3, 6, 9 months
Self-determination theory constructs	Autonomous Self-Regulation (α = .81–.84, r = .38) [78], Competence Scale (α = .88–.95, r = .32–.46) [51, 52, 78].	
Potential moderators/mediators		
Demographics	Age, education level, income	Baseline
HTN/stroke knowledge, health literacy, anthropometrics	Self-report: HTN/stroke knowledge (.70) [54, 55], health literacy (r = .74, .82) [78–80], Height/ weight/girth	Baseline, 3, 6, 9 months
Medication adherence factors	Side effects, adverse events	As reported, and 3, 6, 9 months

^a^Basic psychometric property analyses (e.g., internal consistency, test-retest reliability) will be conducted during first 3 months on scales, which have not been used with Ghanaian stroke patients

*BP* blood pressure, *HTN* hypertension, *IG* Intervention Group

### Outcome measures

#### Feasibility outcomes

This will include recruitment and dropout proportions, protocol adherence, as well as patient and provider satisfaction at 3, 6 and 9 months.

#### Primary outcome measures

This will include percent success in reaching BP control (Table 2) and changes in clinic BP measurements.

**Table 2:** Definition of outcome measures

Variable	Definition
Blood pressure control	Clinic blood pressure control will be defined at resting systolic blood pressure of <140 mmHg and or diastolic blood pressure of <90 mmHg
Medication adherence	Medication adherence will be categorized using Medication Possession Ratios into: Excellent 100 % Good 80–99 % and Poor <80 %.

#### Secondary outcome measures

These will include outcomes such as MPR, Morisky Medication Adherence Scale (MMAS) score and Competence and Autonomous Self-regulation Scale scores.

Table 1 shows all outcome variables, questionnaires to be used and when they will be administered. We will assess self determination theory-related constructs of competence [48, 49], and autonomous self-regulation [50, 51]. We will also assess HTN knowledge [52, 53], medication side effects, stroke knowledge, self-reported medication adherence (MMAS), beliefs and life goals [54, 55] and MPR [56].

### Participant timeline

Algorithms for participant recruitment and follow-up assessments are shown in Fig. 1.

#### Proposed sample size

For this feasibility cluster RCT we will recruit 15 patients per cluster (i.e., physician), 30 patients per group (intervention versus control group) (total *n* = 60). We agree with Kraemer et al. [57] that pilot feasibility studies are deficient in estimating effect size with sufficient accuracy for future study design. To this end we will evaluate the availability of subjects, assess proportions approached/consented, and examine the feasibility of PINGS by evaluating adherence and retention rates. Thus, sample size justification focuses on the precision of estimates rather than the power of statistical tests. We will recruit 30 subjects (15 per physician = cluster) per treatment group based on number of degrees of freedom within each cluster, as suggested by Mead, to obtain reasonable precision for variance estimates [58].

#### Recruitment

A list of eligible patients will be identified from hospital medical records by a research assistant (RA) with the identity of the patients’ doctors not included. The research coordinator (RC) will contact and schedule eligible patients for screening/recruitment and obtain informed consent voluntarily from patients who are interested in the study. Subjects whose SBP averages ≥140 mmHg from the last two readings of the 10-min protocol will have their height, weight and waist circumference measured. A set of questionnaires will be administered to assess study outcome measures (see Table 1). The study site admits an average of 43 stroke patients each month. Based upon the stroke with HTN incidence patterns in the KATH and SMASH pilot work [38–40, 47], we will recruit eight patients (four intervention group patients and four control group patients) per month for enrollment into the study.

#### Allocation and concealment

Randomization of subjects in blocks of four will be conducted by a statistician using a computer-generated random sequence of numbers. Participants who consent to the study will be allocated to either of the two intervention clusters or the two control clusters at the baseline visit using the computer-generated randomization sequence. Each sequence generated will be kept concealed in an envelope which will be opened by the research coordinator in the presence of the consenting eligible study participant at enrollment.

#### Blinding

Physicians who will be assessing primary outcomes and research assistants assessing feasibility and secondary outcomes will remain blinded as to the patients’ group status throughout study (i.e., preintervention, 3-, 6- and 9-month evaluations).

#### Data collection and management

Instruments and questionnaires for data collection are listed in Table 1. Data will be entered into REDCap and, to promote excellent data quality, double data entry by two independent data entry clerks will be performed.

### Planned refinement of the PINGS program

We will refine the PINGS content, delivery format and feedback mechanisms for initial and sustained use, based on subjects’ adherence data, responses to adherence feedback, providers’ responses and other variables that might influence optimal use of the PINGS study [59, 60]. We propose that, over time, positive feedback (including immediate BP feedback, medication adherence feedback, motivational/reinforcement messages) will create sustained behavior change. Although insufficiently powered for formal mediation analyses, we will explore relationships via structural equation modeling using self-determination theory constructs of subjects’ competence (Competence Scale scores) and autonomous regulation (Autonomous Self-Regulation Scale scores) with changes in primary clinical outcomes (changes in BP control, BP and medication adherence), which will help guide further PINGS’ refinement. Providers’ and patients’ attitudes and treatment satisfaction play important roles in the degree to which an intervention is adopted [61–63]. This information will guide how PINGS is presented to the Ghanaian health care community assuming that the feasibility trial and future efficacy/effectiveness RCTs are successful.

### Statistical analyses

#### Feasibility outcomes

We will use 95 % confidence intervals (CIs) for proportions to estimate dichotomous outcomes (e.g., proportion agreeing to participate); adherence to protocol (e.g., use of a medication pill tray, use of a smartphone to send BP measurements, opening of SMS/voice mail messages, and so forth). Frequency distributions will be developed describing reasons for provider and patient protocol nonadherence, dropout and problems encountered such as technology glitches, medication side effects, and so forth. For continuous measures (e.g., patient satisfaction scores), frequency distributions and median and mean responses (with 95 % CIs) will be used. Chi-square tests (categorical measures) and pooled *t* tests (or Wilcoxon rank sum tests) for continuous measures will compare intervention group and SC groups.

#### Primary outcome measures

For these measures, generalized linear mixed models (GLMM) will be used to compare the two groups with group as primary independent variable and percent success reaching BP control (or resting BP changes individually) as the primary dependent variable (Table 2) [64]. GLMM allow for missing data, measurement at different times and take into account the effect of clustering, i.e., correlation of repeated measurements within patients clustered within MD (Medical Doctor) provider. Group and time (baseline, 3, 6 and 9 months) will be fixed effects; demographics and secondary outcome measures (e.g., medication adherence via the MPR) will be adjustment covariates. We will assess changes over time via a time-by-intervention interaction term in multivariable models. We will estimate differences (for means or proportions) in ancillary moderator and mediator measures between the two groups via 95 % CIs as appropriate (e.g., self-efficacy, autonomous regulation, and so forth.). We will estimate differences (via 95 % CI) in proportions and average slopes between intervention versus control groups and evaluate linearity of trajectories as input for future RCT analysis strategy. We will estimate percent success reaching BP control for each subject over the trial (baseline, 3, 6 and 9 months), and the within-subject longitudinal trajectories (e.g., slopes), and then summarize as mean longitudinal trajectory within each treatment group (e.g., mean slopes with 95 % CI). Intracluster correlation coefficient (ICC) and variance estimates will be obtained of efficacy outcomes and covariance structure of the longitudinal scores for determination of sample size (and hence adequate power) for a future RCT. Multiple imputation methods will be used for missing end-of-study outcomes. Dropout rate, both selective and differential, is an important issue. If more than 10 % of data are missing, we will add an intermediate evaluation point in a future RCT to provide more data for use in endpoint imputation.

#### Secondary outcome measures

These are categorical (percent of patients and providers who were adherent) and continuous (e.g., MPR, MMAS score; Competence and Autonomous Self-regulation Scale scores). GLMM will compare the two groups with treatment group as primary independent variable, and outcome measures individually as the dependent variables [64]. Group and time (baseline, 3-, 6- and 9-month visit) will be included as fixed and physician as random effects; health literacy, demographics (age, gender, and so forth) and HTN/stroke knowledge will be adjustment variables. Ninety-five percent CIs will be reported.

### Qualitative studies

After final follow-up (month 9), approximately 10 patients in the intervention group will be randomly selected (5 per physician cluster) to be in a focus group (FG – two will be conducted) of “lived experiences”. Topic areas with probes will cover expectations, experiences, adherence, motivation and advice from family [3, 31, 32]. The two PINGS’ physicians, nurse navigator and other staff involved in the PINGS delivery will also be invited to be in a FG (*n* = approximately 5–8). We will assess attitudes, beliefs, barriers and facilitators for use, as well as feedback on retention, fidelity, impact upon therapeutic inertia and other practice considerations. FGs will be audio-recorded, notes taken, transcribed and imported into NVivo 10.0 for analyses [65]. We will use the constant comparative method of qualitative analysis to code the data [66, 67]. Transcripts will be independently reviewed and coded by two reviewers. Once no new themes emerge, thematic saturation will have been reached [68]. We will then compare and contrast themes from participants and providers using the triangulation approach [69].

### Data Monitoring Committee (DMC)

Our feasibility RCT will not have a DMC due to its preliminary and exploratory nature.

## Discussion

To our knowledge, this would be the first study in SSA to apply synergistic constructs from behavioral and technology application theories and direct guidance from stroke patients, providers and administrators. Secondly, we propose to use real-time adherence measurements of BP data and medication intake to facilitate immediate feedback, and automated motivational/reinforcement messages, all aimed at enhancing self-determination theory constructs of competence (akin to self-efficacy) and autonomous regulation (sustained internally driven motivation). Thirdly, our protocol will give health care providers personally designed automated reports to enable faster changes in medication regimens and earlier sustained BP control.

A feasible and preliminarily effective PINGS’ intervention would lead to a larger, more definitively efficacious RCT powered to look at clinical events, with the potential to reduce HTN-related stroke morbidity, mortality and associated costs in SSA. Second, the proposed task-shifting strategy, which uses nurses to direct BP control, could potentially mitigate the critical shortage of health care workers in the region, and leveraging the high (and rising) mobile phone penetration in the region could integrate care systems and improve patient-provider communication. Third, through our iterative behavioral change theory-guided design process we will be assessing post-trial acceptability, satisfaction, usability, salience and aids/barriers to sustainability among patients, caregivers, providers and other key stakeholders. This information will provide information as to how the PINGS patient- and provider-developed m-Health program in SSA can be refined and eventually disseminated to other locales (assuming that follow-up trials are also effective). Finally, early stage SSA investigators are involved in the PINGS study, which is key for building future research capacity in SSA. Several PINGS team members have successfully mentored junior faculty who have become independently funded investigators and will serve as mentors for the coinvestigators and their research administration staff in Ghana. This will include a progressive exposure to key publications, pod-cast lectures; and direct engagement in trial design, human subject management and execution of a theory-guided m-Health program for addressing chronic disease management. Our collaborators will also receive educational information on the process of developing m-Health research capacity within their hospital network.

Possible limitations to the PINGS study may include the lack of a longer feasibility trial and a lack of intensive lifestyle programs. In response to the first limitation, our selection of a 3-month trial and 6-month follow-up was guided by BP control trials which usually ran for 1–6 months and had no follow-up [20, 21]. A future efficacy RCT will assess if BP control is sustained beyond 9 months. Our approach to the lack of intensive lifestyle programs will be to first focus on how medication adherence and BP monitoring impact BP control without including additional tactics. This is based upon financial costs, response burden and primary practice-based HTN RCTs which found that: (1) adding lifestyle programs to BP monitoring or medication reminder tactics, and so forth, does not necessarily enhance BP control [70] and (2) lifestyle programs are difficult to implement and sustain [71–73]. Also, providers repeatedly request easy-to-implement and efficacious programs. We will have a wealth of data to address important issues not detailed which will guide our future work (e.g., triangulation of the MMAS self-report, pharmacy records (MPR) data to develop effective algorithms for medication adherence measurement). Additional moderator/mediator (understanding they are perhaps underpowered) analyses will be conducted examining the influence of acculturation, health literacy, HTN/stroke knowledge and so forth upon medication adherence and BP control.

### Trial status

Recruitment is anticipated to begin in April 2016.

## Additional file

Additional file 1: SPIRIT checklist. (DOC 121 kb)

## Abbreviations

BP: Blood pressure
BT: Blue-toothed
DBP: Diastolic blood pressure
GLMM: Generalized linear mixed models
HTN: Hypertension
MPR: Medication Possession Ratio
PINGS: Phone-based Interventions under Nurse Guidance after Stroke
RCT: Randomized controlled trial
SBP: Systolic blood pressure
SC: standard care
SMASH: Smart phone Med Adherence Stops hypertension
SMS: Short Message Service
SSA: sub-Saharan Africa
